# LncRNA HCP5 as a potential therapeutic target and prognostic biomarker for various cancers: a meta‑analysis and bioinformatics analysis

**DOI:** 10.1186/s12935-021-02404-x

**Published:** 2021-12-19

**Authors:** Shao-pu Hu, Meng-xue Ge, Lei Gao, Min Jiang, Kai-wen Hu

**Affiliations:** 1 Beijing University of Chinese Medicine, Beijing, 100029 China; 2 Department of Oncology, Dongfang Hospital, Fengtai District, Beijing University of Chinese Medicine, No. 6 Fangxingyuan 1st Block, Beijing, 100078 China; 3 Department of Integrated Management, Dongfang Hospital, Beijing University of Chinese Medicine, Beijing, 100078 China

**Keywords:** lncRNA HCP5, Meta-analysis, Cancer, Prognosis, Bioinformatics

## Abstract

**Background:**

Accumulating studies indicated that dysregulated long non-coding RNA human histocompatibility leukocyte antigen (HLA) Complex P5 (HCP5) may functions as an potential prognostic predictor in multiple cancers. This meta-analysis was performed to systematically collect studies and conduct an evidence-based evaluation of the prognostic role of HCP5 in malignancies.

**Methods:**

Four databases (PubMed, Web of Science, Embase and Cochrane library) were comprehensively retrieved from their initiation date to November 9, 2021. Hazard ratio (HR) or odds ratio (OR) with 95% confidence interval (CI) were used to assess the associations between the expression level of HCP5 and prognosis or clinical characteristics. Moreover, results were validated by Gene Expression Profiling Interactive Analysis 2 (GEPIA2) and the National Genomics Data Center (NGDC). Subsequently, the molecular mechanism of HCP5 was predicted based on MEM and StarBase databases. The study protocol was registered at PROSPERO (ID: CRD42021274208).

**Results:**

9 studies, containing 641 patients, were included in this meta-analysis. Our results revealed that HCP5 overexpression was associated with poor overall survival (OS), tumor type, histological differentiation, and lymph node metastasis in most cancers, but was not associated with age, gender and tumor size; down-regulation of HCP5 was associated with worse OS, advanced tumor stage, positive distal metastasis and lymph node metastasis in skin cutaneous melanoma (SKCM). HCP5 was significantly up-regulated in four cancers and down-regulated in SKCM, which was validated by the GEPIA2 cohort. HCP5 expression in various types of cancer was also verified in NGDC. Further functional prediction revealed that HCP5 may participate in some cancer-related pathways.

**Conclusion:**

There is a significantly association between dysregulation of HCP5 and both prognosis and clinicopathological features in various cancers. HCP5 may be functions as a novel potential prognostic biomarker and therapeutic target in multiple human cancers.

## Introduction

Cancer is the leading cause of death in every country of the world and an important barrier to extending life expectancy [1]. According to the latest global cancer statistics reported in CA cancer journals, an estimated 19.3 million new cancer cases and almost 10.0 million cancer deaths occurred worldwide in 2020 [2]. Despite increasing number of treatment methods for cancer in recent years, the overall prognosis of most cancers remains poor; one of the major causes for this is lack of sensitive and specific biomarker for tumor early diagnosis, most patients are already at an advanced stage when they are initially diagnosed [3]. Early diagnosis and treatment are important to improve the prognosis in cancer patients, therefore, it is of great clinical significance to search novel biomarkers and therapeutic targets of cancer.

Mutations in the non-coding genome were considered to be a major determinant of cancer. Long non-coding RNAs (lncRNAs) are a type of non-coding RNAs with more than 200 nucleotides in length and transcribed by RNA polymerase II [4]. Increasing studies had confirmed that lncRNAs are dysregulated in different cancers, which regulated the progression of malignancies by function as oncogenes or tumor suppressor [5]. Particularly, some lncRNAs have been reported to be markedly associated with the clinicopathological characteristics of cancer patients and may serve as potential therapeutic targets or prognostic biomarkers to predict the clinical outcomes [6, 7].

LncRNA human histocompatibility leukocyte antigen (HLA), complex P5 (HCP5), is primarily found expressed in immune system cells, participating in adaptive and innate immune responses [8, 9]. Recently, several studies proposed that HCP5 could functions as a competitive endogenous RNA (ceRNA) affect the distribution of microRNAs on their targets, thereby participating in the occurrence and progression of tumors. For example, HCP5 expression level was increased in anaplastic thyroid cancer cell lines, HCP5 knockdown inhibited the cell viability and induced apoptosis via sponging miR-128-3p [10]. Furthermore, HCP5 functions as a ceRNA to sponge miR-29b-3p, miR-140-5p, miR-139-5p, and miR-186-5p, which consequently promotes cell growth, metastasis, invasion and epithelial–mesenchymal transition in hepatocellular carcinoma, clear cell renal cell carcinoma, colorectal cancer, or gastric cancer [9, 11–13]. Changes in ceRNA regulation may affect the expression of oncogenes or tumor suppressors, which might provide a potential therapeutic target for cancers [14].

Previous studies have described HCP5 as a cancer promoter in most malignancies, while the expression level of HCP5 has also been found to be downregulated in some tumor tissues. For example, one study found that HCP5 level was decreased in skin cutaneous melanoma, suggesting HCP5 could function as a tumor suppressor and suppresses melanoma development by regulating RARRES3 gene expression via sponging miR-1286 [15]. Clinically, most evidences have showed that HCP5 overexpression is associated with the clinicopathological characteristics and prognosis of various cancers, including gastric cancer [16, 17], non-small cell lung cancer [18], renal cell carcinoma [19] and osteosarcoma [20]. Meanwhile, only one study reported that downregulation of HCP5 was associated with a poor prognosis, advanced tumor stage, positive distal metastasis and lymph node metastasis [15].

Collectively, most studies suggested that HCP5 is more frequently acting as an tumor oncogene in various malignancies, and associated with the prognosis and clinicopathological characteristics. However, results of these previous studies were controversial due to the limitations of the small sample size of single study, the prognostic value of lncRNA HCP5 remains unclear. Therefore, we systematically selected relevant literatures and performed this meta-analysis to conduct an evidence-based evaluation of the prognostic role of lncRNA HCP5 in various cancers.

## Materials and methods

The Preferred Reporting Items for Systematic Reviews and Meta-Analyses (PRISMA) guidelines was used to guide this meta-analysis. This meta-analysis has been registered with PROSPERO (ID: CRD42021274208).

### Literature search and selection

PubMed, Web of Science, Embase and Cochrane library from their initiation date to November 9, 2021 were searched by two investigators (Shaopu Hu and Mengxue Ge). All covered literatures that evaluated the clinicopathological and prognostic value of HCP5 in cancer patients, without language limitation, were collected. The terms of (((((Tumour) OR (tumor)) OR (neoplasms)) OR (cancer)) OR (carcinoma)) AND ((((long non coding RNA HCP5) OR (lncRNA HCP5)) OR (Human histocompatibility leukocyte antigen (HLA) complex P5)) OR (HCP5)) were used as the search strategy.

### Inclusion and exclusion criteria

Inclusion criteria were as the following: (1) studies evaluated the expression level of HCP5 in cancers; (2) patients were divided into low HCP5 expression group and high HCP5 expression group; (3) hazard ratios (HR) and 95% confidence interval (CI) for prognostic indicators were described or could be indirectly calculated according to the survival curves; (4) the relationship between HCP5 and prognosis or clinicopathological features was reported.

Exclusion criteria were as the following: (1) studies evaluated a group of lncRNAs rather than a single HCP5; (2) non-clinical study, editorials, letters, expert opinions, reviews and case reports; (3) necessary data cannot be extracted; (4) prognosis or clinicopathological data from bioinformatics analysis.

### Data extraction and quality assessment

The data of included studies were extracted independently by two reviewers (SH and MG). If there were any divergences, a discussion was performed with another investigator (Kaiwen Hu). The following information were extracted from each study: first author, publication year, region, sample size, tumor type, detection method, outcome, follow-up months, HRs and 95% CIs for OS. We followed the methods of Hu et al. [21]. The quality of included studies was assessed independently by two reviewers (SH and MG). Any divergences were resolved through discussion with a third investigator. The Newcastle–Ottawa Scale (NOS) was applied to assess the quality of all studies. The total scores for different studies ranged from 0 to 9. The study was considered to be high quality if the score was > 6 [22].

### Validation of bioinformatics database

Gene Expression Profiling Interactive Analysis 2 (GEPIA2, http://gepia2.cancer-pku.cn/), which is based on RNA sequencing expression data from the The Cancer Genome Atlas (TCGA) and the GTEx projects [23], was utilized to further verify the expression level and survival analysis of HCP5 in various cancers. One-way ANOVA was used for differential expression analysis. Kaplan–Meier method and log-rank test were used for survival analysis [24]. National Genomics Data Center (NGDC, https://bigd.big.ac.cn) was utilized to further verify the expression level of HCP5 in various cancer types [25].

### Prediction the functions and pathways of HCP5

MEM (http://biit.cs.ut.ee/mem/index.cgi) was used to predict the target genes of HCP5 based on Affymetrix Gene Chip Human Genome U133 Plus 2.0 Array platform. Then, Database for Annotation, visualization and Integrated Discovery (DAVID, https://david.ncifcrf.gov/) was applied to perform GO and KEGG analyses based on the target genes. Moreover, StarBase (http://starbase.sysu.edu.cn/) was utilized to predict the miRNA-HCP5 interactions, with was supported by ago CLIP-seq data. Finally, Cytoscape software (version 3.6.0, https://cytoscape.org/) was used to construct the network of HCP5-target genes and miRNA-HCP5 interactions.

### Statistical analysis

Statistical analysis in this meta-analysis were performed by REVIEW MANAGER 5.3 software, which followed the methods of Hu et al. 2018 [21]. Based on the reported Kaplan–Meier curve in each included study, the HRs and 95% CIs were estimated using the software of Engauge Digitizer 10.0 [21]. The survival results were calculated by log HR and standard error (SE) values. Moreover, the association between HCP5 expression levels and the tumor clinicopathological parameters (age, gender, differentiation, LNM, tumor size and TNM stage) were evaluated by ORs and 95% CIs. The heterogeneity of the eligible studies was evaluated by the Q and I^2^ test. The application of effects model depends on the heterogeneity among the studies. If no significant heterogeneity (I^2^ < 50%, P > 0.1) in the included studies, fixed-effects model was used to analyze the results; while random-effects model was applied for meta-analysis if significant heterogeneity (I^2^ ≥ 50%, P ≤ 0.1) existed in the eligible studies [21]. Funnel plot was used to evaluate the potential publication bias.

## Results

### Literature screening

**Fig. 1:**
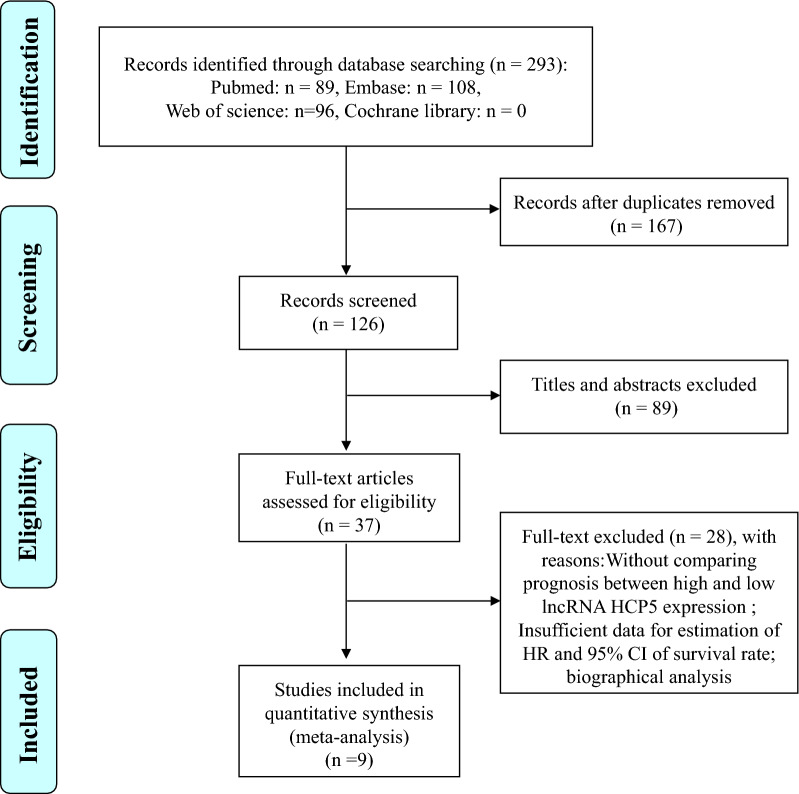
Flow diagram of process for the literature identification and selection

The detailed process of the literature identification and selection was presented in Fig. 1. Initially, a total of 293 publications were retrieved by the search strategy, while 167 of the duplicated articles were excluded. After reviewing the titles and abstracts, 89 literatures were removed because of not cancer related articles or not original research works (reviews, case reports, letters and editorials). After checking the full text of remaining articles, 28 studies were further excluded due to the reason of these studies without comparing the prognosis between high and low HCP5 expression or providing insufficient data for estimation of HR and 95% CI for survival rate or data from biographical analysis. Finally, nine literatures [13, 15–20, 26, 27] coincided with the inclusion criteria were included in the present meta-analysis.

### Study characteristics and quality assessment

The primary characteristics of nine included studies were shown in Table 1. All studies were from China and enrolled a total of 641 patients. The types of cancer in these studies included: non-small cell lung cancer, colorectal cancer, oral squamous cell carcinoma, clear cell renal cell carcinoma, gastric cancer, skin cutaneous melanoma and osteosarcoma. All included studies [13, 15–20, 26, 27] evaluated the association between HCP5 expression levels and overall survival, the follow-up months ranged from 36 to 105. Five studies [13, 15, 16, 19, 26] reported the clinicopathological parameters. The method of all studies to measure the expression of HCP5 was real-time quantitative polymerase chain reaction. The NOS outcome showed all scores were ≥ 6, which suggested that these studies displayed a medium-high quality.

**Table 1:** Characteristics of the included studies

Study	Year	Region	Tumor type	Sample size (low/high)	HR (95% CI)	Outcome	Method	Follow-up months	NOS
Li [18]	2020	China	NSCLC	63 (31/32)	0.44 (0.23, 0.84)	OS	qRT-PCR	60	7
Yang [13]	2019	China	CRC	135 (43/92)	0.36 (0.16, 0.81)	CP, OS	qRT-PCR	60	7
Zhao [26]	2019	China	OSCC	73 (37/36)	0.63 (0.35, 1.13)	CP, OS	qRT-PCR	60	7
Hao [19]	2020	China	ccRCC	66 (33/33)	0.55 (0.28, 1.08)	CP, OS	qRT-PCR	60	7
Liang [17]	2021	China	GC	36 (18/18)	0.47 (0.24, 0.92)	OS	qRT-PCR	60	6
Qin [16]	2021	China	GC	98 (49/49)	0.36 (0.13, 1.00)	CP, OS	qRT-PCR	36	6
Zhang [27]	2020	China	ccRCC	76 (38/38)	0.33 (0.09, 1.19)	OS	qRT-PCR	60	7
Tu [20]	2021	China	OSC	40 (21/19)	0.33 (0.09, 1.21)	OS	qRT-PCR	60	6
Wei [15]	2019	China	SKCM	54 (27/27)	2.97 (1.64, 5.36)	CP, OS	qRT-PCR	105	7

*NSCLC* non-small cell lung cancer, *CRC* colorectal cancer, *OSCC* Oral squamous cell carcinoma, *ccRCC* clear cell renal cell carcinoma, *GC* gastric cancer, *SKCM* skin cutaneous melanoma, *OSC* osteosarcoma, *CP* clinicopathological parameters, *OS* overall survival, *HR* hazard ratios, *CI* confidence intervals, *NA* not available, *NOS* Newcastle–Ottawa Scale

### Association between HCP5 expression levels and OS

**Fig. 2:**
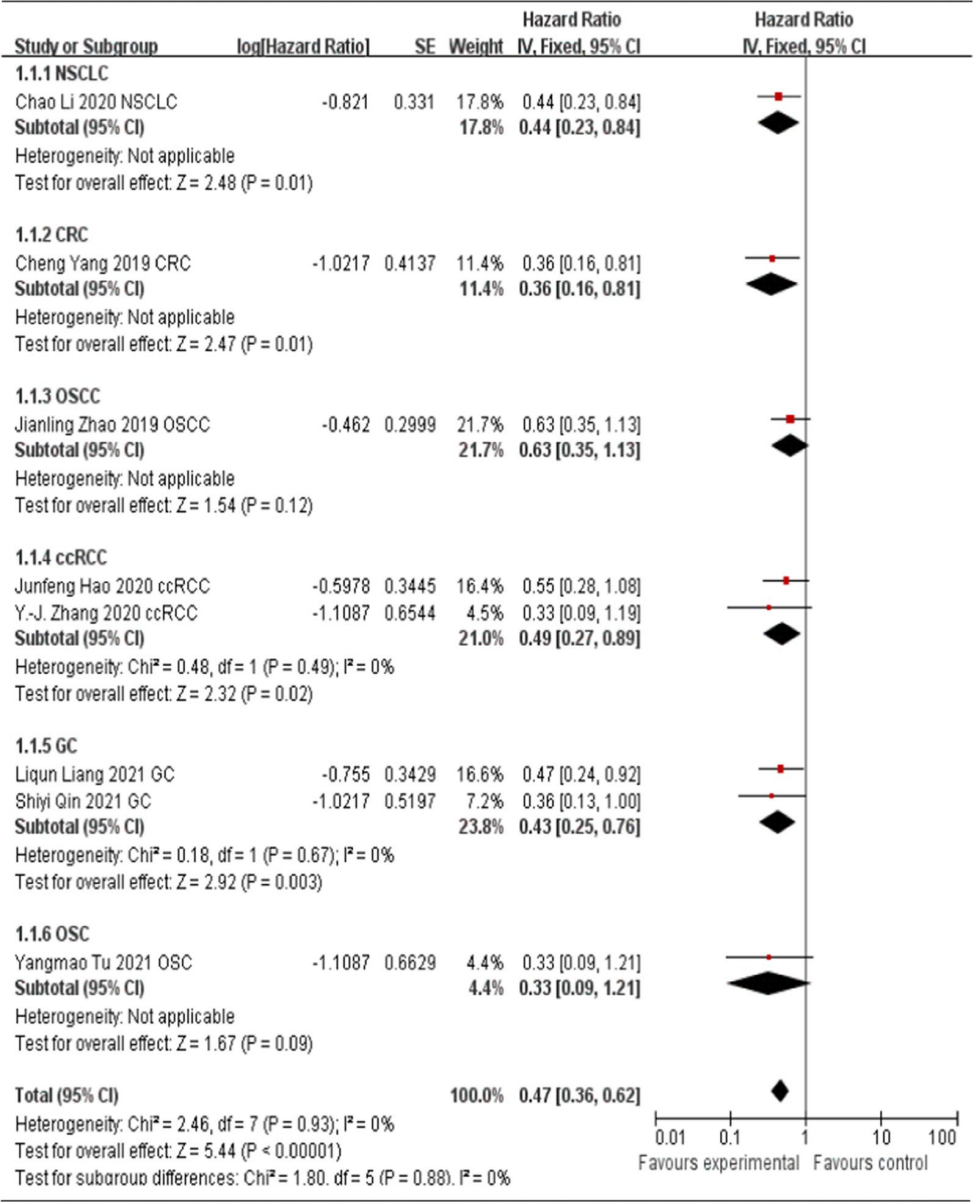
Forest plot for the association between HCP5 expression levels and OS

There were nine studies evaluated the association between HCP5 expression levels and OS in this meta-analysis. Eight [13, 16–20, 26, 27] of them reported that HCP5 was up-regulated in cancer tissues and function as the oncogenes. Therefore, these eight studies were pooled for analysis. As shown in Fig. 2, fixed-effects model was used (I^2^ = 0%, P = 0.93). The pooled HR = 0.47 (95% CI 0.36–0.62, P < 0.00001), which indicated that higher HCP5 expression in the tumor tissues of patients with non-small cell lung cancer, colorectal cancer, oral squamous cell carcinoma, clear cell renal cell carcinoma, gastric cancer and osteosarcoma were associated with a poor OS. Only one study [15] reported that HCP5 function as the tumor suppressor in skin cutaneous melanoma, down-regulation of HCP5 was associated with a poor OS (HR = 2.97, 95% CI 1.64–5.36), advanced tumor stage, positive distal metastasis and lymph node metastasis. Therefore, the data of this study could not be pooled for follow-up analysis.

### Association between HCP5 expression levels and tumor types

**Fig. 3:**
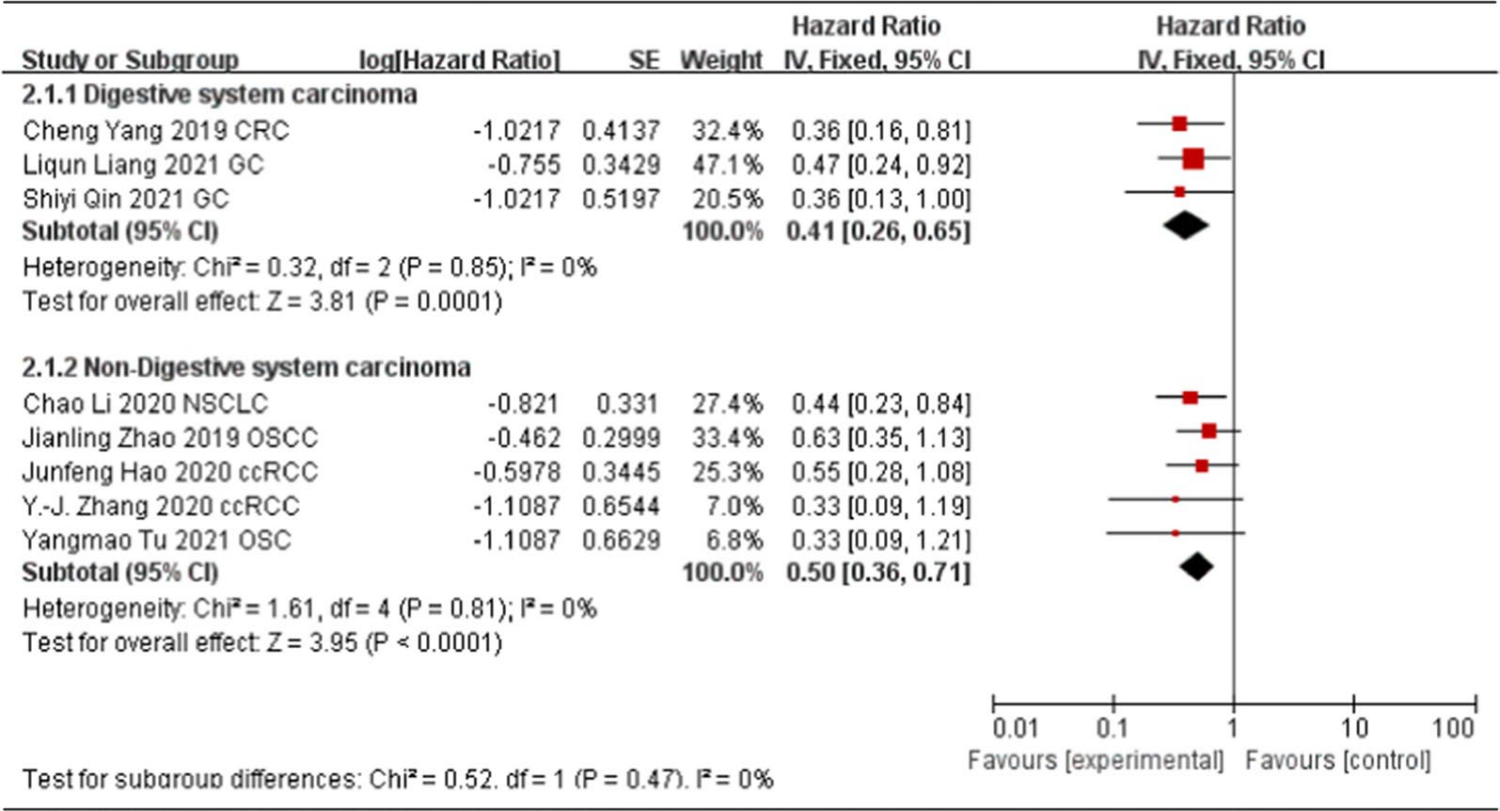
Forest plot for the association between HCP5 expression levels and tumor type

To further explore the potential prognostic value of HCP5, we evaluated the association between up-regulated HCP5 and tumor types. As shown in Fig. 3, three studies [13, 16, 17] explored the digestive system carcinoma and five studies [18–20, 26, 27] explored the non-digestive system carcinoma. The results of the forest plot suggested that regardless of the digestive system carcinoma or non-digestive system carcinoma, up-regulated HCP5 expression in tumor tissues was correlated with poor OS (digestive system carcinoma, HR = 0.41; 95% CI 0.26–0.65, P = 0.0001; non-digestive system carcinoma, HR = 0.50; 95% CI 0.36–0.71, P < 0.0001, Fig. 3).

### Associations between HCP5 expression levels and clinicopathological parameters

**Fig. 4:**
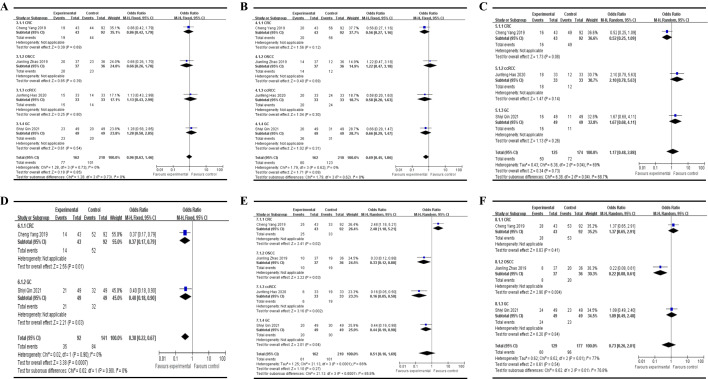
Forest plot for the association between HCP5 expression levels and age (**A**), gender (**B**), tumor size (**C**), tumor differentiation (**D**), Lymph node metastasis (**E**) and TNM stage (**F**)

There were five studies evaluated the association between HCP5 expression levels and clinicopathological parameters in this meta-analysis. One study [15] reported HCP5 function as the tumor suppressor in skin cutaneous melanoma has been described in the above part, we conducted further meta-analysis for other five studies. As shown in Table 2, there were no statistically significant relationships between HCP5 overexpression and age (total OR = 0.96, 95% CI 0.63–1.46, *P* = 0.85, Fixed model, Fig. 4A), gender (total OR = 0.69, 95% CI 0.45–1.06, *P* = 0.09, Fixed model, Fig. 4B) and tumor size (total OR = 1.17, 95% CI 0.48–2.88, *P* = 0.73, Random model, Fig. 4C); However, HCP5 overexpression was statistically correlated with poor histological differentiation in CRC and GC (total OR = 0.38, 95% CI 0.22–0.67, P = 0.0007, fixed model, Fig. 4D), positive lymph node metastasis in CRC (OR = 2.48, 95% CI 1.18–5.21, *P* = 0.02), OSCC (OR = 0.33, 95% CI 0.12–0.88, *P* = 0.03), ccRCC (OR = 0.16, 95% CI 0.05–0.50, *P* = 0.002), and GC (OR = 0.44, 95% CI 0.19–0.98, *P* = 0.04) (Fig. 4E), and advanced TNM stage in OSCC (OR = 0.22, 95% CI 0.08–0.61, *P* = 0.004, Fig. 4F).

**Table 2:** Meta-analysis results for the association between HCP5 expression and clinicopathological characteristics

Characteristics	Cancer type	OR (95% CI)	*P* value	I^2^%	Model
Age (old vs young)	CRC	0.86 (0.42, 1.79)	0.69		
	OSCC	0.66 (0.26, 1.70)	0.39		
	ccRCC	1.13 (0.43, 2.99)	0.80		
	GC	1.28 (0.58, 2.85)	0.54		
	Total	0.96 (0.63, 1.46)	0.85	0	Fixed
Gender (male vs female)	CRC	0.56 (0.27, 1.16)	0.12		
	OSCC	1.22 (0.47, 3.18)	0.69		
	ccRCC	0.58 (0.20, 1.63)	0.30		
	GC	0.66 (0.29, 1.47)	0.31		
	Total	0.69 (0.45, 1.06)	0.09	0	Fixed
Tumor size (large vs small)	CRC	0.52 (0.25, 1.09)	0.08		
	ccRCC	2.10 (0.78, 5.63)	0.14		
	GC	1.67 (0.68, 4.11)	0.26		
	Total	1.17 (0.48, 2.88)	0.73	69	Random
Differentiation (poor vs well)	CRC	0.37 (0.17, 0.79)	0.01*		
	GC	0.40 (0.18, 0.90)	0.03*		
	Total	0.38 (0.22, 0.67)	0.0007*	0	Fixed
Lymph node metastasis (yes vs no)	CRC	2.48 (1.18, 5.21)	0.02*		
	OSCC	0.33 (0.12, 0.88)	0.03*		
	ccRCC	0.16 (0.05, 0.50)	0.002*		
	GC	0.44 (0.19, 0.98)	0.04*		
	Total	0.51 (0.16, 1.69)	0.27	86	Random
TNM stage (III–IV vs I–II)	CRC	1.37 (0.65, 2.91)	0.41		
	OSCC	0.22 (0.08, 0.61)	0.004*		
	GC	1.09 (0.49, 2.40)	0.84		
	Total	0.73 (0.26, 2.01)	0.54	77	Random

*OR* odds ratio, *CI* confidence interval, *CRC* colorectal cancer, *OSCC* Oral squamous cell carcinoma, *ccRCC* clear cell renal cell carcinoma, *GC* gastric cancer

*P < 0.05

### Publication bias

**Fig. 5:**
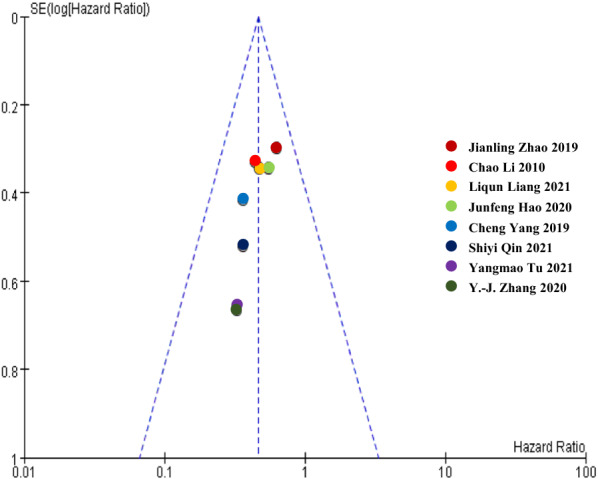
Funnel plots for the association between HCP5 expression levels and OS

A funnel plot has been made to analyze whether our results were influenced by the potential publication bias. Studies reported the association between up-regulated HCP5 expression levels and OS were chosen for analysis, as most of the studies measured it. A total of eight studies [13, 16–20, 26, 27] were analyzed, the results showed that there was no obvious publication bias in the research (Fig. 5).

### Validation results of HCP5 in public databases

**Fig. 6:**
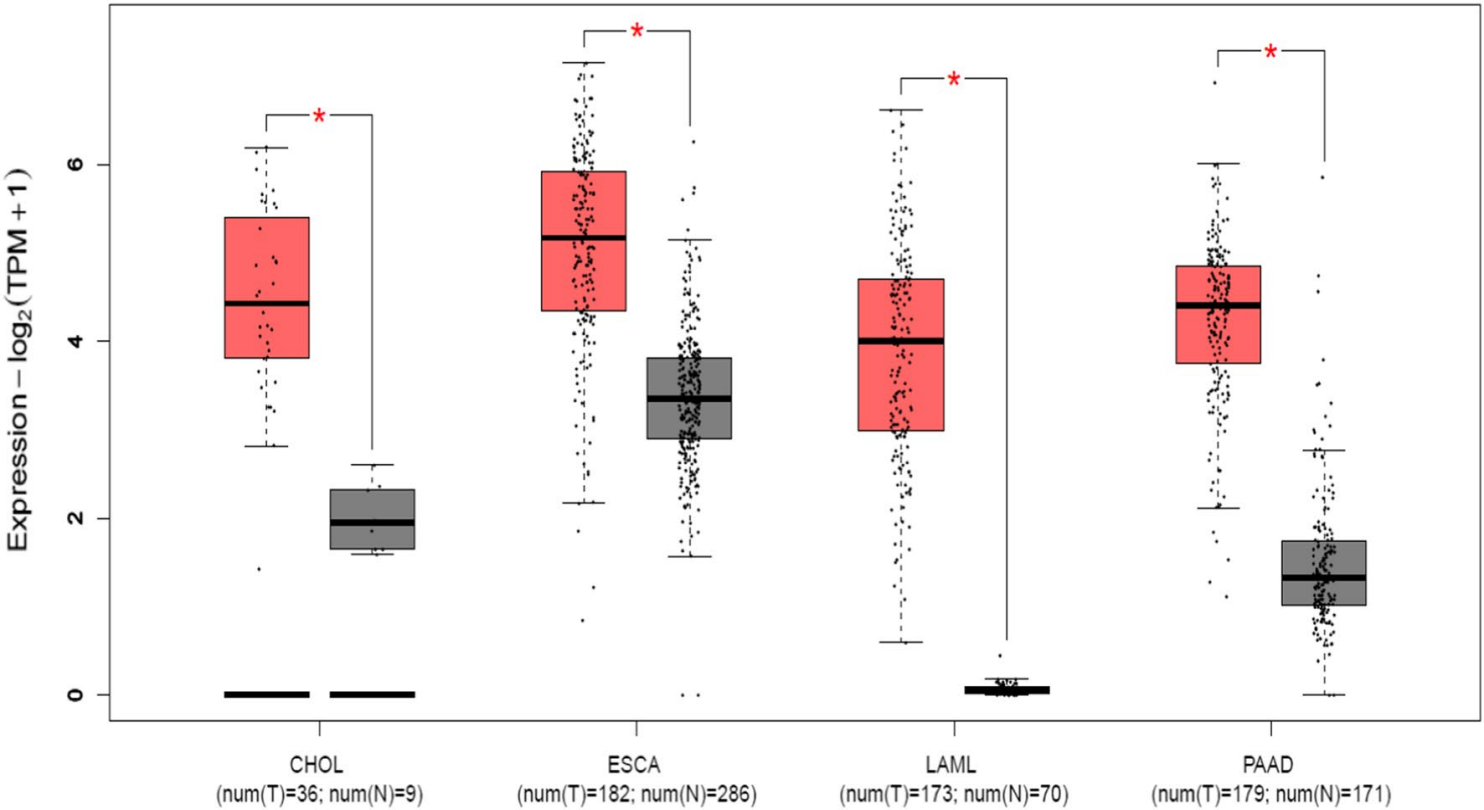
The expression levels of HCP5 in four types of cancer tissues and normal tissues in GEPIA2 cohort. Red box plots, HCP5 expression level in cancer tissues; grey box plots, HCP5 expression level in normal tissues; *log_2_FC| > 1 and P < 0.01

**Fig. 7:**
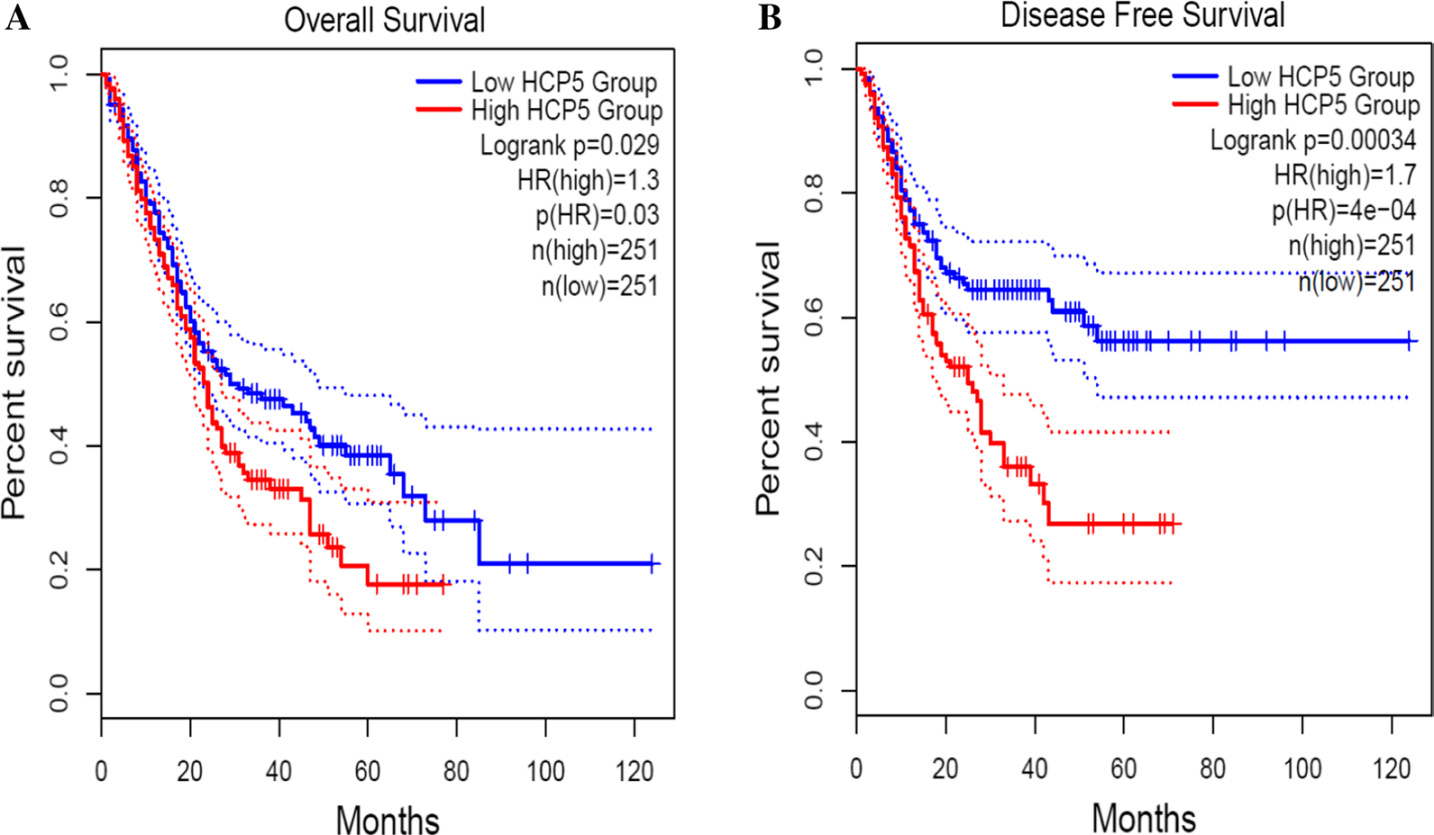
Overall survival plots (**A**) and Disease free survival plots (**B**) of HCP5 in GEPIA2 cohort, including CHOL, ESCA, LAML and PAAD (n = 502)

To further verify our results, we used GEPIA 2 to evaluate the expression level and survival analysis of HCP5 in various cancers. The results indicated that HCP5 was up-regulated in most cancers, including cholangio carcinoma (CHOL), esophageal carcinoma (ESCA), acute myeloid leukemia (LAML) and pancreatic adenocarcinoma (PAAD) (|log_2_FC| Cutoff: 1, P-value Cutoff: 0.01, Fig. 6). Moreover, we merged the expression and prognosis data for four type of cancer, including CHOL, ESCA, LAML and PAAD. As shown in Fig. 7, 502 patients were grouped into high (n = 251) and low (n = 251) HCP5 level groups with the median expression level of HCP5 in four cancer types as the cutoff value. The HCP5 high expression group had shorter OS and DFS than the HCP5 low expression group, confirming that up-regulated HCP5 is correlated with poor OS in most human cancers.

**Fig. 8:**
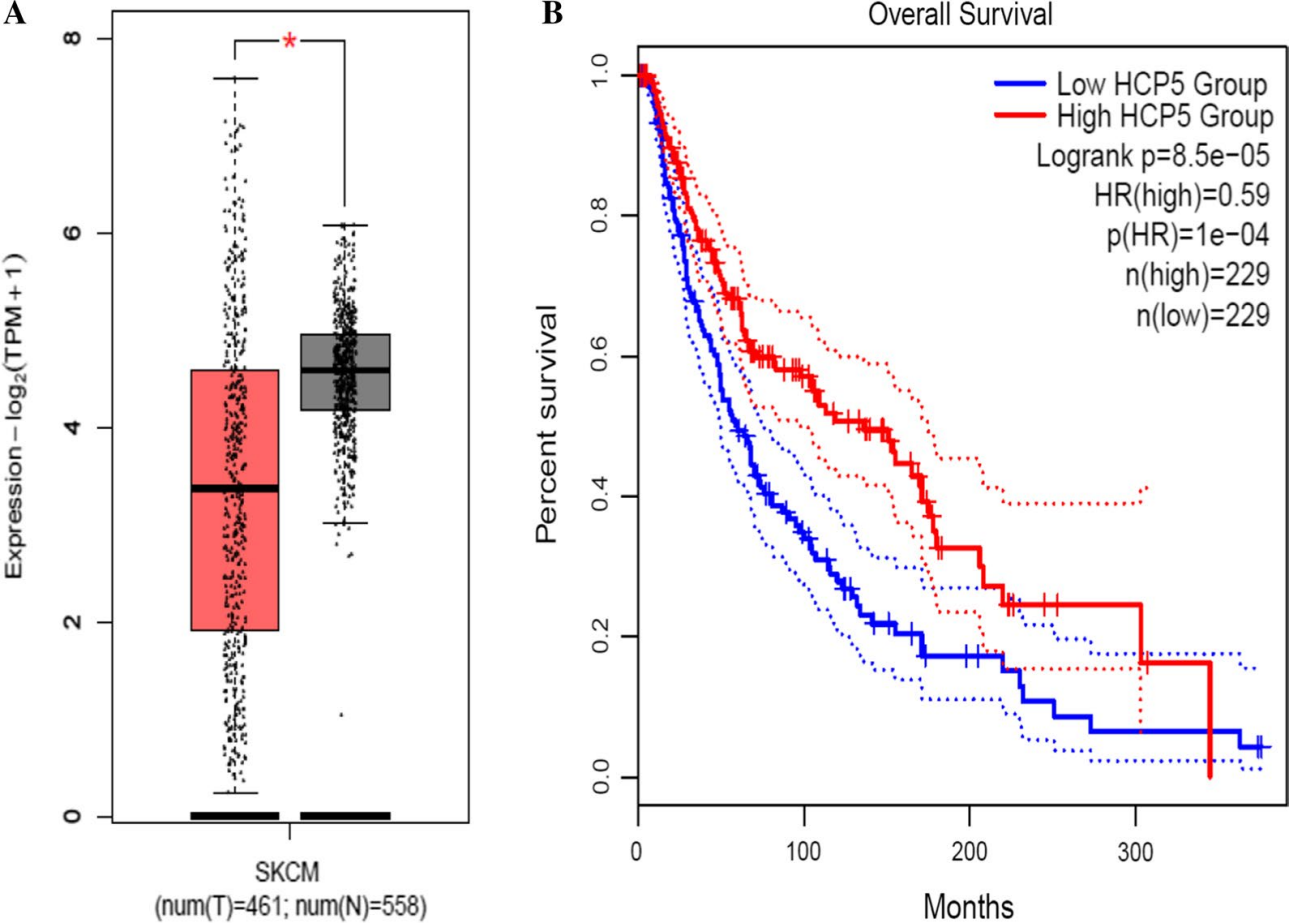
The expression levels (**A**) and Overall survival plots (**B**) of HCP5 in SKCM in GEPIA2 cohort

Meanwhile, we evaluated the expression level and survival analysis of HCP5 in skin cutaneous melanoma (SKCM). As shown in Fig. 8, HCP5 was down-regulated in SKCM, HCP5 high expression group had longer OS than the HCP5 low expression group. This result is consistent with the reported literature, confirming that down-regulated HCP5 is correlated with well OS in SKCM.

NGDC was used to further validate the expression of HCP5 in various cancers. Ten types of cancer (breast cancer, cervical cancer, colon cancer, esophageal tumor, gastric cancer, liver tumor, serous ovarian cancer, bladder cancer, ovarian cancer and prostate cancer) with 27 datasets were collected. As shown in Table 3, HCP5 was more frequently over-expressed in multiple cancer tissues compared with these normal tissues, and the differences were statistically significant (P < 0.05). Regrettably, the expression of HCP5 in SKCM could not be further validated via NGDC since lack SKCM related datasets.

**Table 3:** Differential expression of HCP5 in various cancers

Tumor Type	GSE ID	Sample size		Expression	P-value
		Tumor	Normal		
Breast cancer	GSE42568	104	17	High	0.0004
Triple-negative breast cancer	GSE76250	165	33	High	0.0001
	GSE65194	55	11	High	0.0037
	GSE65212	55	11	High	0.0037
Cervical cancer	GSE29570	45	17	High	0.0000
	GSE63678	5	5	High	0.0007
	GSE67522	20	22	High	0.0270
	GSE39001	43	12	High	0.0376
Colon cancer	GSE28000	81	34	High	0.0152
	GSE84984	7	6	High	0.0152
Esophageal tumor	GSE29001	21	24	High	0.0000
	GSE5364	16	13	High	0.0053
Gastric cancer	GSE63089	45	45	High	0.0002
	GSE49515	3	10	High	0.0004
	GSE36076	3	9	High	0.0006
	GSE58828	3	3	High	0.0040
	GSE56807	5	5	High	0.0051
Liver tumor	GSE14520	225	220	High	0.0000
	GSE11819	8	8	High	0.0000
Serous ovarian cancer	GSE36668	4	4	High	0.0016
	GSE16708	17	9	High	0.0026
Bladder cancer	GSE89006	4	4	Low	0.0000
Ovarian cancer	GSE52037	10	10	Low	0.0001
	GSE18520	53	10	Low	0.0003
Prostate cancer	GSE29079	47	48	Low	0.0000
	GSE38241	18	21	Low	0.0000
	GSE17951	109	45	Low	0.0006

### Prediction results of the functions and pathways of HCP5

**Fig. 9:**
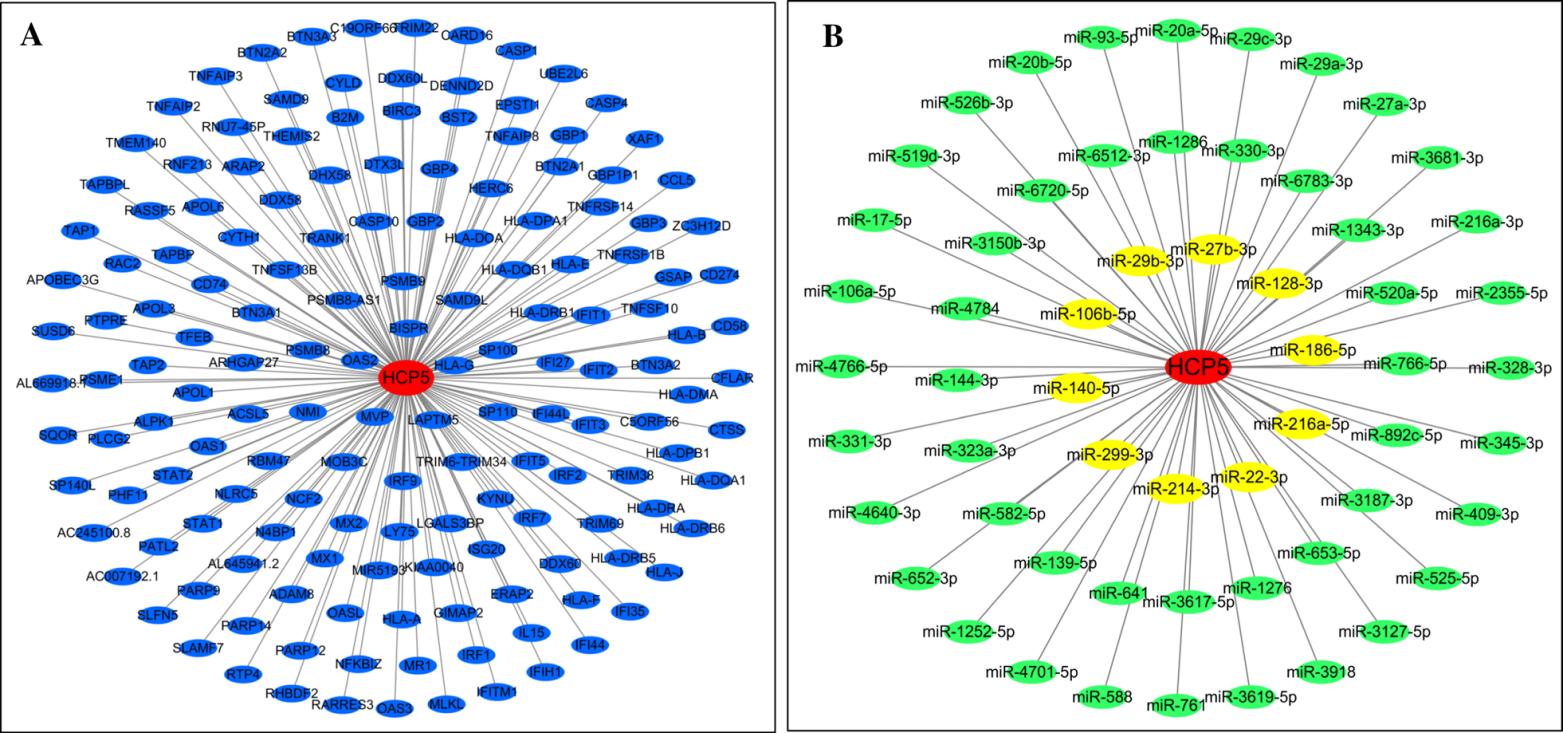
Network analysis between HCP5 and target genes (**A**), miRNA-HCP5 interactions (**B**). Red circle, lncRNA HCP5; Blue circle, target genes of HCP5; Green circle, miRNAs that interacts with HCP5; Yellow circle, miRNAs that interact with HCP5 have been validated

Based on MEM database, a total of 157 target genes of HCP5 were selected and collected to perform the GO and KEGG pathway analyses (Fig. 9A). The most strongly enriched GO terms were identified as follows: immune response, defense response to virus, peptide antigen binding and integral component of lumenal side of endoplasmic reticulum membrane (Table 4). Besides, the KEGG pathway analysis result confirmed that these co-expressed genes were significantly involved in Cell adhesion molecules (CAMs), TNF signaling pathway, NF-kappa B signaling pathway, Apoptosis and Viral carcinogenesis (Table 5). Altogether, these results revealed that HCP5 main participate in the biological mechanism of cancer and autoimmune disease. Moreover, a total of 58 miRNAs were predicted via StarBase, which may exist interactions with HCP5 (Fig. 9B). Ten miRNAs (miR-27b-3p, miR-29b-3p, miR-128-3p, miR-106b-5p, miR-186-5p, miR-140-5p, miR-216a-5p, miR-299-3p, miR-22-3p and miR-213-3p) have been reported, additional studies are needed to explore the potential mechanisms of other miRNAs-HCP5 interactions.

**Table 4:** Top five enrichment GO terms (BP, CC and MF) of the potential genes of HCP5

GO ID	Term	Ontology	Count	P-value
GO:0060337	Type I interferon signaling pathway	BP	29	3.90E−43
GO:0060333	Interferon-gamma-mediated signaling pathway	BP	28	1.57E−39
GO:0051607	Defense response to virus	BP	26	1.89E−25
GO:0006955	Immune response	BP	31	1.04E−20
GO:0009615	Response to virus	BP	20	1.20E−20
GO:0042605	Peptide antigen binding	MF	13	1.12E−18
GO:0032395	MHC class II receptor activity	MF	8	1.09E−11
GO:0003725	Double-stranded RNA binding	MF	8	5.45E−07
GO:0046978	TAP1 binding	MF	4	1.99E−06
GO:0001730	2ʹ-5ʹ-oligoadenylate synthetase activity	MF	4	1.99E−06
GO:0071556	Integral component of lumenal side of endoplasmic reticulum membrane	CC	14	9.17E−21
GO:0012507	ER to Golgi transport vesicle membrane	CC	14	7.32E−17
GO:0042613	MHC class II protein complex	CC	11	2.64E−16
GO:0042612	MHC class I protein complex	CC	8	3.94E−13
GO:0030658	Transport vesicle membrane	CC	8	1.27E−08

*GO* Gene Ontology, *BP* biological process, *CC* cellular component, *MF* molecular function

**Table 5:** KEGG pathway enrichment analysis of the potential genes of HCP5

KEGG ID	Term	Count	P value	Genes
hsa04145	Phagosome	18	9.74E−15	HLA-DRB5, NCF2, HLA-B, TAP2, TAP1, HLA-A, HLA-F, HLA-G, CTSS, HLA-DMA, HLA-DPB1, HLA-DRA, HLA-DOA, HLA-DQA1, HLA-DRB1, HLA-DPA1, HLA-DQB1, HLA-E
hsa04514	Cell adhesion molecules (CAMs)	16	1.31E−12	CD274, HLA-DRB5, HLA-B, HLA-A, HLA-F, HLA-G, HLA-E, HLA-DMA, HLA-DPB1, HLA-DRA, CD58, HLA-DOA, HLA-DQA1, HLA-DRB1, HLA-DPA1, HLA-DQB1
hsa04668	TNF signaling pathway	8	4.89E−05	MLKL, IL15, CASP10, CCL5, TNFAIP3,CFLAR, TNFRSF1B, BIRC3
hsa04064	NF-kappa B signaling pathway	6	0.001138	DDX58, PLCG2, TNFAIP3, CFLAR,TNFSF13B, BIRC3
hsa05203	Viral carcinogenesis	8	0.002532	SP100, IRF7, HLA-B, HLA-A, HLA-F,HLA-G, IRF9, HLA-E
hsa04210	Apoptosis	4	0.018872	CASP10, TNFSF10, CFLAR, BIRC3
hsa04144	Endocytosis	7	0.022316	HLA-B, ARAP2, HLA-A, HLA-F, HLA-G,CYTH1, HLA-E

*KEGG* Kyoto Encyclopedia of Genes and Genomes

### Functional mechanisms of HCP5 in various cancers

The known functional mechanisms of HCP5 in various cancers were summarized in Table 6. Briefly elaborated as follows:

- In thyroid carcinoma, HCP5 promoted the proliferation, migration, invasiveness and angiogenic ability of follicular thyroid carcinoma cells via sponging miR-22-3p, miR-186-5p, miR-216a-5p and activating ST6GAL2. In addition, HCP5 knockdown reduced cell viability, while elevated apoptotic rate via sponging miR-128-3p in anaplastic thyroid cancer [10, 14].
- In bladder cancer, HCP5 upregulation could promote cell invasion and migration via sponging miR-29b-3p [28].
- In triple negative breast cancer, HCP5 as a ceRNA to regulate BIRC3 by sponging miR-219a-5p, thereby promoting cancer progression. In addition, overexpression of HCP5 promoted cisplatin resistance by inhibiting PTEN expression [29, 30].
- In cervical cancer, HCP5 promoted the development of cervical cancer through increasing MACC1 expression by microRNA-15a adsorption [31].
- In clear cell renal cell carcinoma, HCP5 promoted proliferation and metastasis of clear cell renal cell carcinoma via targeting miR-140-5p/IGF1R pathway [12].
- In colon cancer, HCP5 enhanced cell proliferation and migration of colon cancer cells by inhibiting the AP1G1 expression and activating the PI3K/AKT pathway [32].
- In colorectal cancer, HCP5 contributes to epithelial–mesenchymal transition in colorectal cancer through HCP5/miR-139-5p/ZEB1 axis. In addition, HCP5 knockdown inhibited viabilities, migration and invasion, while inducing apoptosis by miR-299-3p/PFN1/AKT axis [13, 33].
- In cutaneous squamous cell carcinoma, HCP5 promoted autophagy and reduced apoptosis by competitively bind to miR-138-5p to regulate EZH2 [34].
- In esophageal squamous cell carcinoma, HCP5 promoted cellular activities via modulating the miR-139-5p/PDE4A pathway and stimulating the PI3K/AKT/mTOR signaling pathway [35].
- In gastric cancer, HCP5 was highly expressed in gastric cancer cell lines, HCP5 silencing inhibited cell proliferation, migration, invasion, and promoted cell apoptosis via regulation of miR-299-3p/SMAD5 axis [36]. Meanwhile, HCP5 induced EMT processes via the miR-186-5p/WNT5A axis under hypoxia [9], and contributes to cisplatin resistance through miR-519d/HMGA1 and miR-128/HMGA2 axis [17, 37].
- In pancreatic cancer, HCP5 silencing inhibited proliferation, migration, and invasion by downregulating CDK8 via sponging miR-140-5p [38].

**Table 6:** Summary of functional characterization of HCP5 in various cancers

Cancers	Expression	Related genes	Role	References
Thyroid carcinoma	Upregulated	miR-22-3p, miR-186-5p, miR-216a-5p, ST6GAL2	Oncogene	[14]
Anaplastic thyroid cancer	Upregulated	miR-128-3p	Oncogene	[10]
Bladder cancer	Upregulated	miR-29b-3p, HMGB1	Oncogene	[28]
Breast cancer	Upregulated	miR-219a-5p, BIRC3	Oncogene	[29]
TNBC	Upregulated	PTEN	Oncogene	[30]
Cervical cancer	Upregulated	miR-15a, MACC1	Oncogene	[31]
ccRCC	Upregulated	miR-140-5p, IGF1R	Oncogene	[12]
Colon cancer	Upregulated	PI3K/AKT/AP1G1	Oncogene	[32]
Colorectal cancer	Upregulated	miR-299-3p, PFN1/AKT	Oncogene	[33]
Colorectal cancer	Upregulated	miR-139-5p, ZEB1	Oncogene	[13]
CSCC	Upregulated	miR-138-5p, EZH2	Oncogene	[34]
ESCC	Upregulated	miR-139-5p/PDE4A PI3K/AKT/mTOR	Oncogene	[35]
Gastric cancer	Upregulated	miR-299-3p, SMAD5	Oncogene	[36]
Gastric cancer	Upregulated	miR-186-5p, WNT5A	Oncogene	[9]
Gastric cancer	Upregulated	miR-519d, HMGA1	Oncogene	[37]
Gastric cancer	Upregulated	miR-128, HMGA2	Oncogene	[17]
Glioma	Upregulated	miR-139, RUNX1	Oncogene	[42]
Glioma	Upregulated	miR-128	Oncogene	[43]
Hepatocellular carcinoma	Upregulated	miR-29b-3p, DNMT3A	Oncogene	[11]
Large B-cell lymphoma	Upregulated	miR-27b-3p, MET	Oncogene	[44]
Lung adenocarcinoma	Upregulated	miR-203, SNAI	Oncogene	[45]
NSCLC	Upregulated	miR-320, Survivin	Oncogene	[18]
Multiple myeloma	Upregulated	miR-128-3p, PLAGL2	Oncogene	[46]
Neuroblastoma	Upregulated	miR-186-5p, MAP3K2	Oncogene	[47]
OSCC	Upregulated	miR-140-5p, SOX4	Oncogene	[48]
Osteosarcoma	Upregulated	miR-101, EPHA7	Oncogene	[20]
Ovarian cancer	Upregulated	miR-525-5p, PRC1	Oncogene	[49]
Pancreatic cancer	Upregulated	miR-214-3p, HDGF	Oncogene	[8]
Pancreatic cancer	Upregulated	miR-140-5p, CDK8	Oncogene	[38]
Prostate cancer	Upregulated	miR-4656, CEMIP	Oncogene	[50]
Renal cell carcinoma	Upregulated	miR-214-3p, MAPK1	Oncogene	[19]
Retinoblastoma	Upregulated	miR-3619-5p, HDAC9	Oncogene	[51]
Skin cutaneous melanoma	Downregulated	miR-12, RARRES3	Suppressor	[15]

*TNBC* triple-negative breast cancer, *ccRCC* clear cell renal cell carcinoma, *CSCC* cutaneous squamous cell carcinoma, *ESCC* esophageal squamous cell carcinoma, *NSCLC* non-small cell lung cancer cells, *OSCC* oral squamous cell carcinoma

## Discussion

With the rapid development of high-throughput sequencing technology, the field of lncRNAs has attracted extensive attention. Countless studies have revealed that lncRNAs could participate in various chemical and biological processes, such as chromosome remodeling, transcription, cancer metastasis and posttranscriptional processing [39]. Accumulating studies have shown that lncRNAs were abnormally expressed in various cancers, and function as oncogene or tumor suppressor based on these expression level, participate in the initiation and progression of cancer. Previous meta-analyses have indicated that DLX6-AS1 [40], PVT1 [41], SNHG15 [22] and GHET1 [24] are related to the clinicopathological features and prognosis of various cancers. These evidences indicated that lncRNA may be a specific prognostic biomarker and therapeutic target for cancer.

HCP5, a promising novel cancer-related lncRNA, numerous studies have revealed that HCP5 was dysregulated in various cancers, and that HCP5 has the potential to become a diagnostic biomarker and therapeutic target. Li et al. found that HCP5 was significantly overexpression in NSCLC tissues, and the overall survival rate of NSCLC patients in high HCP5 group was significantly lower [18]. Yang et al. reported that HCP5 expression was higher in colorectal cancer tissues than in adjacent normal tissue, and that HCP5 overexpression was correlated with low TNM stage, poor differentiation and low tumor depth invasion [13]. Zhao et al. found that HCP5 expression was increased in oral squamous cell carcinoma tissues compared with adjacent normal tissues, and that high expression of HCP5 was closely associated with lymph node metastasis, advanced TNM stage and the unfavorable overall survival in oral squamous cell carcinoma patients [26]. Hao et al. [19] showed that HCP5 expression was enhanced in neoplasm tissues of renal cell carcinoma patients, and that high HCP5 was related to Fuhrman neoplasm grade and lymphatic metastasis. Tu et al. [20] demonstrated HCP5 was up-regulated both in osteosarcoma tissues and cell lines and high expression of HCP5 was associated to low survival in osteosarcoma patients.

This is the first meta-analysis to systematically collect studies and to evaluate the potential functions of HCP5 as therapeutic target and prognostic biomarker for human cancers. We found that HCP5 overexpression increased the risk of shorter overall survival in most cancers (non-small cell lung cancer, colorectal cancer, oral squamous cell carcinoma, clear cell renal cell carcinoma, gastric cancer and osteosarcoma). Moreover, the relationship between HCP5 expression levels and clinical attributes has been analyzed according to the specific cancer types. The results suggest that there were no statistically significant relationships between HCP5 overexpression and age, gender and tumor size. However, HCP5 overexpression was statistically correlated with poor histological differentiation in CRC and GC, positive lymph node metastasis in CRC, OSCC, ccRCC and GC, and advanced TNM stage in OSCC. In addition, only one study reported that down-regulation of HCP5 was associated with worse OS, advanced tumor stage, positive distal metastasis and lymph node metastasis in skin cutaneous melanoma, the number of study restricted the prognostic meta-analysis. Furthermore, we used two public databases (GEPIA 2 and NGDC) to validate HCP5 expression level in various cancers. The results revealed that HCP5 was significantly up-regulated in four cancers (CHOL, ESCA, LAML and PAAD), and that strongly associated with poor prognosis; HCP5 was down-regulated in SKCM, and that HCP5 high expression group had longer OS than the HCP5 low expression group. These results are consistent with the findings presented in this study.

In sum up, dysregulation of HCP5 expression was closely related to tumor prognosis, thus may be a potential prognostic biomarker in cancers. Then, we used bioinformatics databases to predict the function mechanism of HCP5. According to GO and KEGG analyses, we found that the most strongly enriched functional terms were immune response, defense response to virus, peptide antigen binding and integral component of lumenal side of endoplasmic reticulum membrane. Also, the HCP5 target genes were significantly related to TNF signaling pathway, NF-kappa B signaling pathway and Apoptosis. Up to now, no studies were found on HCP5 and TNF signaling pathway or NF-kappa B signaling pathway. Moreover, a total of 58 miRNAs-HCP5 interactions were predicted, which could provide reference for future molecular mechanism research of HCP5.

Nonetheless, several limitations in the present study should be emphasized. First, our meta analysis included only nine studies, limited sample sizes, and all from China, there was the possibility of selection bias for positive results in our research of the literatures; Second, only one study reported the association between down-regulated of HCP5 with both prognosis and clinicopathological features in SKCM, the reliability of the conclusion may be limited. Third, no standard cut-off value to measure the expression level of HCP5, and the data of HRs and 95% CIs in some studies were extracted by the software of Engauge Digitizer, which might generate statistical errors. Finally, the prediction results of the functions and pathways of HCP5 needs to be validated further in experiments.

## Conclusion

In summary, based on current reported literature, there is significantly association between dysregulation of HCP5 and both prognosis and advanced clinicopathological features in most cancers. HCP5 may be functions as a novel potential prognostic biomarker and therapeutic target in multiple human cancers. However, there were still several limitations in our study, high-quality and multicenter studies are still needed to confirm these conclusions.

## Data Availability

All data are included in this article.

## Abbreviations

lncRNA: Long noncoding RNA
HCP5: Human histocompatibility leukocyte antigen (HLA) complex P5
HR: Hazard ratio
OR: Odds ratio
NSCLC: Non-small cell lung cancer
CRC: Colorectal cancer
OSCC: Oral squamous cell carcinoma
ccRCC: Clear cell renal cell carcinoma
GC: Gastric cancer
SKCM: Skin cutaneous melanoma
OSC: Osteosarcoma
CP: Clinicopathological parameters
OS: Overall survival
CI: Confidence intervals
NOS: Newcastle–Ottawa Scale
GO: Gene Ontology
BP: Biological process
CC: Cellular component
MF: Molecular function
